# Exploratory analysis of the effect of helminth infection on the immunogenicity and efficacy of the asexual blood-stage malaria vaccine candidate GMZ2

**DOI:** 10.1371/journal.pntd.0009361

**Published:** 2021-06-01

**Authors:** Odilon Nouatin, Juliana Boex Mengue, Jean Claude Dejon-Agobé, Rolf Fendel, Javier Ibáñez, Ulysse Ateba Ngoa, Jean Ronald Edoa, Bayodé Roméo Adégbité, Yabo Josiane Honkpéhédji, Jeannot Fréjus Zinsou, Aurore Bouyoukou Hounkpatin, Kabirou Moutairou, Andreas Homoet, Meral Esen, Andrea Kreidenweiss, Stephen L. Hoffman, Michael Theisen, Adrian J. F. Luty, Bertrand Lell, Selidji Todagbe Agnandji, Ghyslain Mombo-Ngoma, Michael Ramharter, Peter Kremsner, Benjamin Mordmüller, Ayôla Akim Adegnika

**Affiliations:** 1 Centre de Recherches Médicales de Lambaréné, Lambaréné, Gabon; 2 Institut für Tropenmedizin, Universität Tübingen, Tübingen, Germany; 3 Département de Biochimie et de Biologie Cellulaire, Faculté des Sciences et Techniques, Université d’Abomey-Calavi, Cotonou, Bénin; 4 Center of Tropical Medicine and Travel Medicine, Department of Infectious Diseases, Amsterdam University Medical Centers, Amsterdam Infection & Immunity, Amsterdam Public Health, University of Amsterdam, Amsterdam, The Netherlands; 5 German Center for Infection Research (DZIF), Partner Site Tübingen, Tübingen, Germany; 6 Fondation pour la Recherche Scientifique, Cotonou, Bénin; 7 Department of Parasitology, Leiden University Medical Centre (LUMC), Leiden, The Netherlands; 8 Sanaria, Inc., Rockville, Maryland, United States of America; 9 Department of Congenital Disorders, Statens Serum Institut, Copenhagen, Denmark; 10 Centre for Medical Parasitology at Department of International Health, Immunology and Microbiology, University of Copenhagen, Copenhagen, Denmark; 11 Department of Infectious Diseases, Copenhagen University Hospital, Rigshospitalet, Copenhagen, Denmark; 12 Centre d’Etude et de Recherche sur le Paludisme Associé à la Grossesse et à l’Enfance, Calavi, Bénin; 13 Université de Paris, MERIT, IRD, Paris, France; 14 Department of Medicine I, Division of Infectious Diseases and Tropical Medicine, Medical University of Vienna, Vienna, Austria; 15 Department of Tropical Medicine, Bernhard Nocht Institute for Tropical Medicine & I, Department of Medicine, University Medical Centre, Hamburg-Eppendorf, Hamburg, Germany; 16 Department of Medical Microbiology, Radboud University Medical Center, Nijmegen, the Netherlands; Stanford University, UNITED STATES

## Abstract

**Background:**

Helminths can modulate the host immune response to *Plasmodium falciparum* and can therefore affect the risk of clinical malaria. We assessed here the effect of helminth infections on both the immunogenicity and efficacy of the GMZ2 malaria vaccine candidate, a recombinant protein consisting of conserved domains of GLURP and MSP3, two asexual blood-stage antigens of *P*. *falciparum*. Controlled human malaria infection (CHMI) was used to assess the efficacy of the vaccine.

**Methodology:**

In a randomized, double-blind Phase I clinical trial, fifty, healthy, lifelong malaria-exposed adult volunteers received three doses of GMZ2 adjuvanted with either Cationic Adjuvant Formulation (CAF) 01 or Alhydrogel, or a control vaccine (Rabies) on days (D) 0, D28 and D56, followed by direct venous inoculation (DVI) of 3,200 *P*. *falciparum* sporozoites (PfSPZ Challenge) approximately 13 weeks after last vaccination to assess vaccine efficacy. Participants were followed-up on a daily basis with clinical examinations and thick blood smears to monitor *P*. *falciparum* parasitemia for 35 days. Malaria was defined as the presence of *P*. *falciparum* parasites in the blood associated with at least one symptom that can be associated to malaria over 35 days following DVI of PfSPZ Challenge. Soil-transmitted helminth (STH) infection was assessed by microscopy and by polymerase chain reaction (PCR) on stool, and *Schistosoma* infection was assessed by microscopy on urine. Participants were considered as infected if positive for any helminth either by PCR and/or microscopy at D0 and/or at D84 (Helm+) and were classified as mono-infection or co-infection. Total vaccine-specific IgG concentrations assessed on D84 were analysed as immunogenicity outcome.

**Main findings:**

The helminth in mono-infection, particularly *Schistosoma haematobium* and STH were significantly associated with earlier malaria episodes following CHMI, while no association was found in case of coinfection. In further analyses, the anti-GMZ2 IgG concentration on D84 was significantly higher in the *S*. *haematobium*-infected and significantly lower in the *Strongyloides stercoralis-*infected groups, compared to helminth-negative volunteers. Interesting, in the absence of helminth infection, a high anti-GMZ2 IgG concentration on D84 was significantly associated with protection against malaria.

**Conclusions:**

Our results suggest that helminth infection may reduce naturally acquired and vaccine-induced protection against malaria. Vaccine-specific antibody concentrations on D84 may be associated with protection in participants with no helminth infection. These results suggest that helminth infection affect malaria vaccine immunogenicity and efficacy in helminth endemic countries.

## Author summary

Helminths, mainly because of their immune regulatory effects, are able to impact the response induced by vaccines. In the context of clinical trial designs that measure accrual of natural infections during follow up or outcome of controlled human malaria infection (CHMI), their effect on vaccine efficacy can be measured. Indeed, most of such clinical trials on malaria vaccine candidates conducted in Africa, especially where the prevalence of helminths is high, have shown a certain limit in their efficacy and immunogenicity, as compared to results observed in European and U.S volunteers. The present analysis assessed the effect of helminths on GMZ2, a malaria vaccine candidate. We found a high level of anti-GMZ2 antibodies among volunteers not infected with helminths and protected against CHMI, indicating efficacy of the candidate vaccine in this population. We found a species-dependent effect of helminths on the level of post-immunization GMZ2-specific IgG concentration, and an association of helminths with an early onset of malaria in CHMI. Our findings reveal that helminths are associated with immunogenicity and may decrease the protective effect of antibodies induced by vaccination. Helminth infection status shall be determined when measuring the immunogenicity and efficacy of malaria vaccine candidates in helminth endemic countries.

## Introduction

Helminth infections remain widespread and cause important neglected tropical diseases. The clinical presentations include anemia, malnutrition, developmental deficiencies causing significant morbidity and mortality [1]. Blood flukes like *Schistosoma haematobium* and the group of soil-transmitted helminths (STH) including *Ascaris lumbricoides*, hookworm, *Trichuris trichiura* and *Strongyloides stercoralis* are most prevalent in developing countries, particularly in sub-Saharan Africa [2], where malaria endemicity is high. In areas of co-endemicity, individuals frequently harbour these infections concomitantly. Several studies have already highlighted the role of helminth infections on the modulation of immune responses directed against *Plasmodium falciparum* antigens or against vaccine antigens, although with contradictory findings. Some studies reported a down-modulating effect of *S*. *haematobium* infection on anti-*P*. *falciparum* immune responses such as negative association between the intensity of *S*. *haematobium* infection and IgG1, IgG3 and IgG4 antibody subclass levels directed to malarial total schizont extract [3]. *S*. *haematobium* infection has also been shown to affect specific IgG1 directed to *P*. *falciparum* (Pf) MSP1 and GLURP [4], or to affect the anti-Pfs48/45 IgG level [5]. By contrast, other studies have reported protection against malaria due to a Th2-enriched environment associated with *S*. *haematobium* infection [6], or anti-malarial protective antibody responses favored by *S*. *haematobium-P*. *falciparum* coinfection [7]. With regard to STH infection, some authors have reported an association with an increased risk of clinical malaria [8] while other studies ruled out an effect of these helminth infections on the course of *Plasmodium* infection [9,10]. A recent study carried out on school-age children in rural area of Gabon showed an increased risk of *P*. *falciparum* infection due to STH in schistosomiasis-positive children [11]. Many studies have also been conducted to assess the effect of helminth infection on several commonly used vaccines [12–18], but little is known about their interaction with malaria vaccine candidates [19–22].

GMZ2, a malaria vaccine candidate, is a recombinant fusion protein with fragments of *P*. *falciparum* GLURP and MSP3 [23] that showed good immunogenicity when formulated with aluminum hydroxide (alum) [24–26] or Cationic Adjuvant Formulation 01 (CAF01) adjuvant [27]. Importantly, pre-school aged children vaccinated with GMZ2-alum and infected with *T*. *trichiura* had low vaccine-specific IgG responses compared to their non-infected counterparts or those infected with *A*. *lumbricoides* [20]. That study clearly suggested that helminth infections affected GMZ2 vaccination-induced responses. We therefore hypothesized that helminth infection would negatively affect the immunogenicity and efficacy of GMZ2. Our study focused on *S*. *haematobium* mainly. Indeed, the study area is endemic for *S*. *haematobium* [5,11,28,29], although *S*. *mansoni* infections may also rarely occur [30]. In the present exploratory analysis, our main objective was to assess the effect of *S*. *haematobium* and STH on the time of the occurrence of malaria episodes after CHMI. In addition to that, we also assessed the effect of that helminth on the immunogenicity and the efficacy of GMZ2 when adjuvanted with either CAF01 or alum in Gabonese adults.

## Methods

### Ethics statement

The original study was approved by the Comité National d’Ethique de la Recherche of Gabon, under the reference N°004/2015/SG/P. The trial was performed according to the International Council for Harmonization of Technical Requirements for Pharmaceuticals for Human Use Good Clinical Practice guidelines and the Declaration of Helsinki. Written informed consent was obtained from all participants included in the study.

### Study area

The original study was conducted at the Centre de Recherches Médicales de Lambaréné (CERMEL), Gabon (April 2015 –November 2015). Lambaréné is a semi-urban town, an area in which malaria [31] and other parasitic infections particularly *S*. *haematobium* and STH [28] are endemic.

### Study design

The detailed design of the main study is described elsewhere [27]. Briefly, eligible participants were randomized to receive three doses, 4-weeks apart (Day 0, Day 28, Day 56) of either 30μg or 100μg of GMZ2-CAF01, 100μg GMZ2-Alhydrogel (alum), or control rabies vaccine (Verorab, Sanofi Pasteur). Vaccine efficacy was assessed using standardized controlled human malaria infection (CHMI) by direct venous inoculation (DVI) of aseptic, purified, cryopreserved 3,200 *P*. *falciparum* sporozoites (PfSPZ Challenge, Sanaria Inc.) approximately 13 weeks after the last immunization (on Day 56), and participants were followed-up on a daily basis with clinical and parasitological (thick blood smears for malaria) examinations for 35 days (S1 Fig). Malaria was defined as the presence of *P*. *falciparum* parasites associated with at least one malaria-attributable symptom over 35 days following DVI of PfSPZ Challenge. Typical malaria symptoms include fever, tachycardia, chills, rigor, sweats, headache, anorexia, nausea, vomiting, myalgia, arthralgia, chest pain, low back pain, abdominal pain and fatigue. CHMI results in 34 volunteers showed that five participants were fully protected (no parasitaemia and no malaria symptoms), fourteen controlled parasitaemia (low oscillating parasitemia and no symptoms) and fifteen had developed malaria (monotone increase of parasitemia with symptoms). The proportion of participants who developed malaria and the time to develop malaria were similar in all vaccinated groups [27]. Participants were treated immediately when they met the malaria case definition. For participants without malaria, follow-up was censored at 35 days after PfSPZ Challenge inoculation (C+35) and treatment was administered irrespective of malaria status on that day. *P*. *falciparum* infection was treated with artemether-lumefantrine as the first line treatment. Urine samples were collected at D0 and at D84 on three consecutive days whereas stool samples were collected once at D0 and once at D84 (S1 Fig). Urine and stool were examined for the presence of *Schistosoma* eggs and STH eggs or larvae, respectively. An aliquot of samples was frozen for DNA extraction and was processed after D84. Participants were considered as helminth infected if found positive on D0 and/or on D84 by any helminth species. All infected participants detected by microscopy were treated for helminth infection after D84 according to local guidelines.

### Laboratory analysis

#### GMZ2-specific IgG concentration measurements

Specific anti-GMZ2 IgG as well as those directed to MSP3 and GLURP were assessed in sera by Enzyme Linked ImmunoSorbent assay (ELISA), as described elsewhere [27]. Briefly, the micro titration plates (Nunc) were coated with 100 μl of GMZ2, 100 μl of GLURP or 100 μl of MSP3 diluted in PBS, and plates were covered and incubated overnight at 4°C. Following plates washing, 150 μl of the blocking solution were added in each well and plates were incubated for 1 hour at room temperature. The plates were washed and the diluted serum samples were added in each well plate, followed by incubation for 2 hours at room temperature. After washing, the plates were incubated with 100 μl of peroxidase-conjugated goat anti-human IgG diluted to 1/65000 with the dilution solution, then incubated for 1 hour at room temperature. The plates were washed and 100 μl of the color solution (TMB One) were added, incubated for 20 min at temperature room in the dark, followed by addition of 100 μl of 0.2M sulfuric acid. The plates were immediately read at an absorbance of 450 nm and a reference at 620 nm using a microplate reader (Thermo Multiskan, Model 355).

### Helminth detection by microscopy

The Kato Katz enrichment and microscopy was used to detect eggs of hookworms, *A*. *lumbricoides*, and *T*. *trichiura* in stool samples, and coproculture was performed to detect larvae of hookworms and *S*. *stercoralis* as described elsewhere [28,32,33]. Urine (around 10 mL) filtration followed by microscopy was used to detect the presence of *S*. *haematobium* eggs in urine [29]. Presence of microfilariae of *Loa loa* and *Mansonella perstans* in blood was assessed using the leuco-concentration technique [34].

### DNA extraction, amplification, and detection

#### Parasite DNA from stool

DNA extraction, amplification, and detection Parasite DNA from stool was isolated and amplified following procedures previously described with minor modifications [35–37]. For the isolation of DNA, 100 mg of stool was suspended in 200 μL of phosphate-buffered saline (Sigma-Aldrich) with 2% polyvinylpolypyrolidone (Sigma-Aldrich) [36], followed by homogenization and bead-beating performed in a 2 ml tube containing Lysing Matrix E (MP Biomedicals). Suspended feces were frozen at -70°C for 30 min. After thawing, suspensions were treated with sodium dodecyl sulphate-proteinase K for 2 h at 55°C [37] and DNA was extracted with the QIAamp DNA mini kit (QIAgen) according to the manufacturer’s instruction. During the isolation an internal extraction control, 10^3^ PFU/ml phocine herpes virus 1 (PhHV-1) was added within the isolation lysis buffer in each sample [37]. Previously described PCR primer and probe sequences [38,39] were used for the amplification of *A*. *lumbricoides*, *S*. *stercoralis*, *Necator americanus* and *T*. *trichiura*. Those species were the most detected by microscopy in our study and are known to be most prevalent in the study area [40]. Primer and probe concentrations were optimized for the assay (S1 and S2 Tables). Amplification conditions were 15 min at 95°C followed by 45 cycles of 15 s at 95°C, 30 s at 60°C and 30 s at 72°C respectively. Amplification and detection of fluorescence was done on a LightCycler 480 (Roche) machine. Cq values ≤ 39 were considered as positive. No DNA extraction was done on urine samples.

### Statistical analyses

Data were exported in R version 3.4.2 for statistical analysis. Graphs were made using GraphPad Prism Version 6 and R. Study participants were divided in two main groups; those found to be uninfected with helminths by both qPCR and microscopy formed the helminth negative (Helm-) group, and those shown to be infected with helminths either by qPCR and/or by microscopy formed the helminth positive (Helm+) group. In order to assess the effect of individual helminth species, subgroups were formed based on the respective mono-infections i.e. *S*. *haematobium* (Sh+) and *S*. *stercoralis* (Str+). Due to the very low number of cases, and as no considerable difference in concentration of anti-GMZ2 antibodies was found between these both STH, *T*. *trichiura* and hookworm infections were merged into the third Tt+/Hw+ sub-study group. The remaining study participants constituted the coinfection (CoInf+) sub-study group as they were infected with more than one parasite. The Mann-Whitney non-parametric test was used to compare characteristics and the haematological profiles of the study population, while linear regression was used for univariable and multivariable analyses to compare specific IgG levels between study groups, using baseline vaccine-specific total IgG concentration as variable of adjustment. The unpaired t test was performed to compare the specific IgG concentration between each combination of two groups after stratification of the helminth infected group, following by ANOVA test with Holm-Sidak’s multiple comparisons. A Kaplan-Meier curve and Log-rank tests were used to assess the time to malaria episodes between study groups and subgroups. All analyses were considered exploratory. The tests were considered statistically significant for a *p-value* less than 0.05.

## Results

### Participants flow and characteristics of the study population

**Fig 1:**
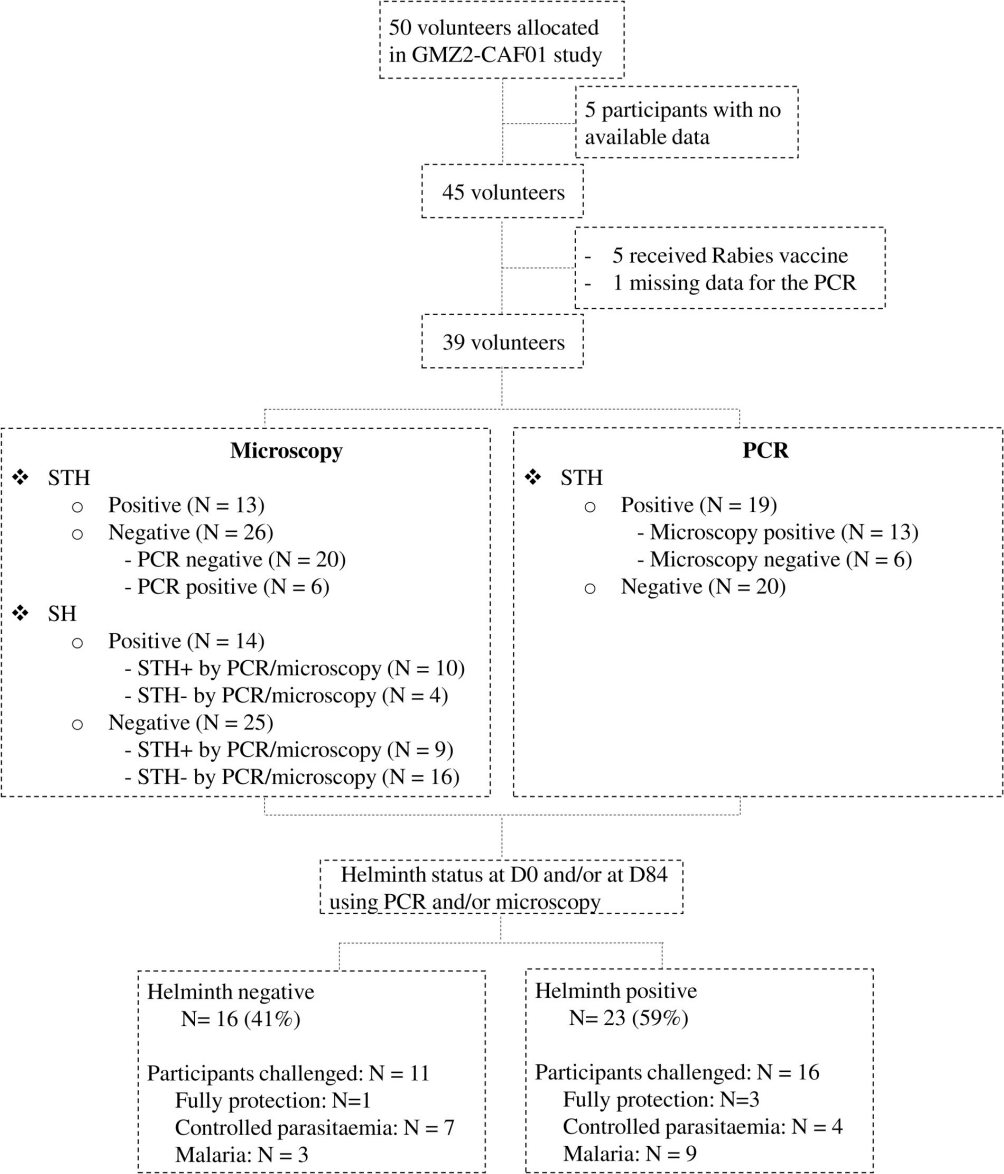
Flow chart of the study participants. PCR: Polymerase chain reaction STH: Soil-transmitted helminth SH: *Schistosoma haematobium* N = number

Of the forty-five (45) participants from the fifty (50) included in the clinical trial and for whom all data are available, five (5) were excluded from analysis as they received the control vaccine (Verorab) and one further patient was excluded due to missing data for the PCR on stool samples for STH detection. Data on thirty-nine participants are therefore considered for analysis (Fig 1). Of them, thirteen (13) were infected with at least one STH species (Hookworm, *A*. *lumbricoides*, *T*. *trichiura*, *S*. *stercoralis*) as determined by the presence of eggs or larvae in stool using microscopy, while twenty-six (26) were considered uninfected. Using the PCR method, a total of nineteen (19) participants were found to be infected with at least one species of STH, including 6 of those for whom microscopy was negative, while the twenty (20) other participants were uninfected (Fig 1).

Assessing participants’ schistosomiasis status using urine filtration, a total of fourteen (14) participants had *Schistosoma* eggs in urine, including ten (10) participants also infected with STH using microscopy and/or PCR, and twenty-five (25) participants had no *Schistosoma* eggs, including nine (9) participants infected with STH using microscopy and/or PCR. No *Schistosoma* eggs were found in stool samples, and no filarial infection was detected in the study population. In summary, 16 (41%) participants were classified as uninfected (Helm-), and 23 (59%) were classified as infected (Helm+) (Fig 1).

Among the participants included in the Helm+ group, 4 had *S*. *haematobium* alone, 4 had *S*. *stercoralis*, 2 had *T*. *trichiura* and 1 had hookworm. As detailed in S3 Table, 12 participants were infected with more than one helminth species and were included in a CoInf group, with *S*. *haematobium* being the most prevalent species (34%), followed by *T*. *trichiura* (28%), Hookworm (21%), *S*. *stercoralis* (10%) and *A*. *lumbricoides* (7%), respectively. All 39 participants included in this analysis were men with a median age (interquartile range) of 23 (5.5) years. Compared to the Helm- group, the hemoglobin level was lower in the Helm+ group (*p-value* = 0.02) while the levels of white blood cells (*p-value* = 0.0008), lymphocytes (*p-value* = 0.001), eosinophils (*p-value* = 0.0006) and basophils (*p-value* = 0.001) were increased (Table 1).

**Table 1:** Characteristics and hematological profile of the study population on D84 with regard to helminth status.

	Helminth negative	Helminth positive	*p*-value*
**N (%)**	16 (41)	23 (59)	-
**Age (years)**	25 (10)	22 (4)	0.06
**BMI (kg/m**^**2**^**)**	22.40 (3.05)	22.50 (2.5)	0.79
**Hb (g/dL)**	14.7 (1.4)	13.6 (1.6)	**0.02**
**WBC (x10**^**3**^**/mm**^**3**^**)**	4.25 (1.37)	5.70 (1.55)	**0.0008**
**Lymphocytes (x10**^**3**^**/mm**^**3**^**)**	2.01 (0.49)	2.42 (0.68)	**0.001**
**Neutrophils (x10**^**3**^**/mm**^**3**^**)**	1.97 (1.18)	2.62 (1.76)	0.05
**Eosinophils (x10**^**3**^**/mm**^**3**^**)**	0.07 (1.18)	0.33 (0.82)	**0.0006**
**Basophils (x10**^**3**^**/mm**^**3**^**)**	0.04 (0.03)	0.06 (0.04)	**0.001**

BMI = Body mass index, Hb = Hemoglobin, WBC = White blood cells.

* Mann-Whitney test. Data are median (interquartile range)

### Helminth infection and vaccine-specific IgG concentration

**Fig 2:**
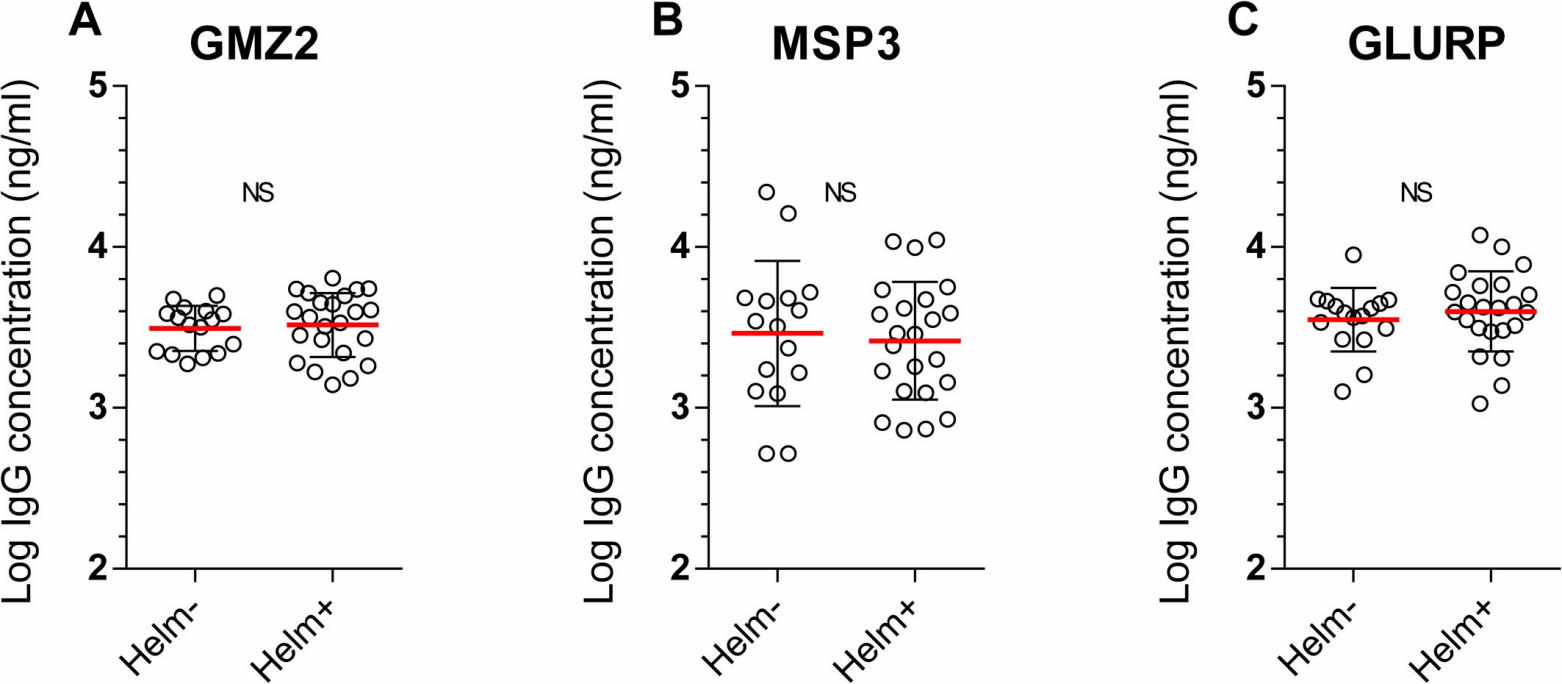
Post immunization antibody concentration at D84 in helminth uninfected and infected groups (Helm- and Helm+). Fig 2 illustrates the log of GMZ2-specific (A), MSP3-specific (B), GLURP-specific (C) IgG concentration at D84 in helminth negative (Helm-, n = 16) and helminth positive (Helm+, n = 23) groups. Comparisons were performed by univariate and multivariate linear regression adjusted for baseline data as independent covariable. The graphs are mean ± standard deviation. Statistical significance was set for p value below 0.05. *p<0.05, **p<0.01, ***p<0.001, ****p<0.0001. NS = Non-significant.

As shown elsewhere [27] and confirmed here, immunization with GMZ2 induced a high levels of anti-GMZ2, anti-MSP3 and anti-GLURP IgG antibodies. The differences in antibody levels between D0 and D84 are presented in S2 Fig. Here, we compared the antibody concentration on D84 between Helm- and Helm+ groups, as well as with infected subgroups. No statistically significant relationship was observed between helminth status and level of anti-GMZ2 IgG (*p-value* = 0.49), anti-MSP3 (*p-value* = 0.18) and anti-GLURP IgG (*p-value* = 0.30) concentration (Fig 2A–2C). No significant relationship was observed when applying multivariate analyses.

As presented in Table 2, we found at univariate analysis no relationship between helminths status and the level of the three antibody concentrations measured and this in the three vaccine groups.

**Table 2:** Comparison of antibody log concentrations mean (±SD) between helminth infection groups at D84 stratified by study vaccination groups.

	100μg GMZ2-Alum			30μg GMZ2-CAF01			100μg GMZ2-CAF01		
	Helm- (n = 07)	Helm+ (n = 04)	*p-value*	Helm- (n = 03)	Helm+ (n = 05)	*p-value*	Helm- (n = 06)	Helm+ (n = 14)	*p-value*
**Anti-GMZ2 IgG**	3.47 (**±**0.15)	3.64 (**±**0.11)	0.09	3.51 (**±**0.14)	3.53 (**±**0.22)	0.90	3.50 (**±**0.13)	3.47 (**±**0.20)	0.70
**Anti-MSP3 IgG**	3.52 (**±**0.45)	3.34 (**±**0.36)	0.52	3.60 (**±**0.65)	3.19 (**±**0.25)	0.23	3.32 (**±**0.38)	3.51 (**±**0.38)	0.30
**Anti-GLURP IgG**	3.52 (**±**0.16)	353 (**±**0.11)	0.91	3.39 (**±**0.27)	3.46 (**±**0.29)	0.73	3.64 (**±**0.16)	3.66 (**±**0.25)	0.88

Helm+ = positive for helminth infection

Helm- = negative for helminth infection

**Fig 3:**
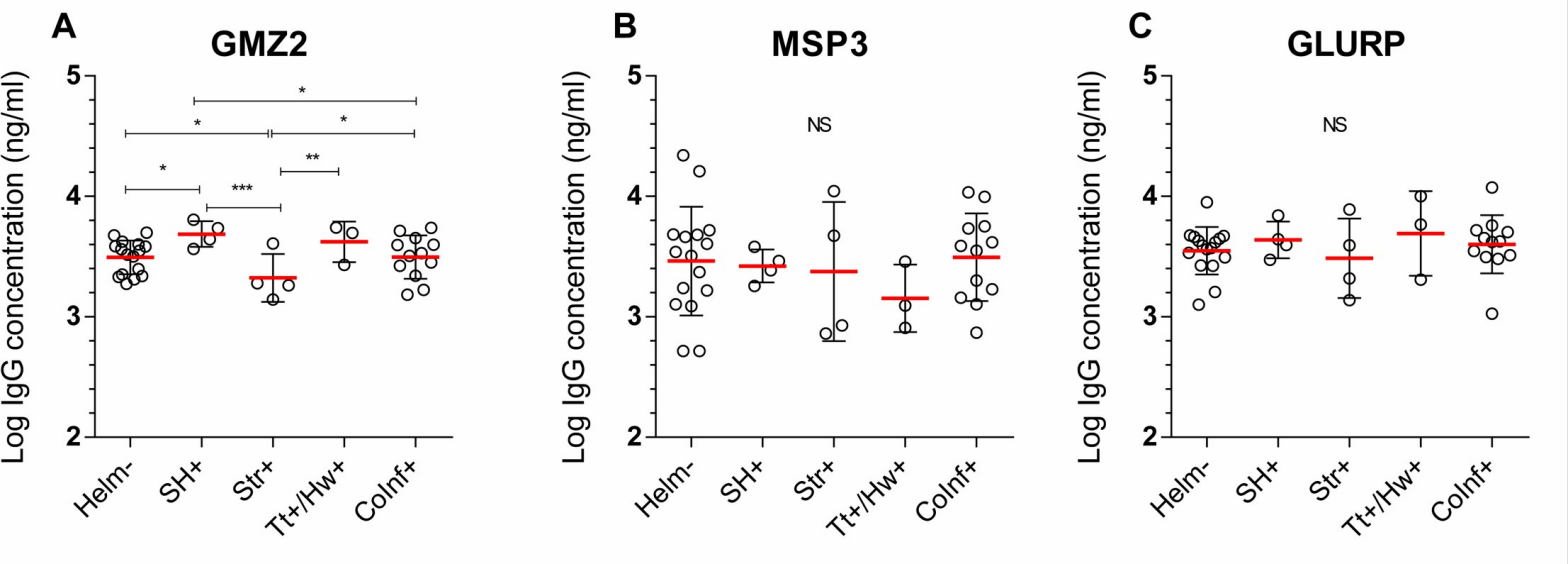
Post immunization antibody concentration at D84 in Helm- and Helm+ subgroups. Fig 3 illustrates the log of specific-GMZ2 (A), specific-MSP3 (B), specific GLURP (C) IgG concentration at D84 in Helm- and mono-infected by *Schistosoma haematobium* (Sh+, n = 4), those mono-infected with Strongyloides (Str+, n = 4), those mono-infected with *Trichuris trichiura* or hookworm (Tt+/Hw+, n = 3), and those coinfected by at least two different helminths (Coinf+, n = 12). The comparison was performed by multivariate linear regression adjusted for baseline vaccine-specific total IgG concentration as independent covariable. The graphs show mean ± standard deviation. Statistical significance was set for p value below 0.05 and is indicated when statistically significant. *p<0.05, **p<0.01, ***p<0.001. NS = Non-significant.

Considering the sub-study groups, we found a relationship between *S*. *haematobium* status (*p-value* = 0.01), *S*. *stercoralis* (*p-value* = 0.03) status and vaccine-specific IgG concentration on D84. Compared to anti-GMZ2 IgG concentration on D84 in those uninfected (mean log concentration ±SD: 3.49 ±0.14), participants infected with *S*. *haematobium* alone presented a higher level of anti-GMZ2 IgG (3.69 ±0.10), while those infected with *S*. *stercoralis* alone had lower anti-GMZ2 IgG levels (3.32 ±0.19) (Fig 3A). Additionally, a significantly lower level of anti-GMZ2 IgG was observed in Str+ individuals compared with the Sh+ individuals (*p-value* = 0.0008), Tt+/Hw+ individuals (3.62 ±0.16, *p-value* = 0.003) and CoInf+ individuals (3.49 ±0.17, *p-value* = 0.03) (Fig 3A). No significant differences between the groups were observed when applying a correction for multiple comparison. In addition, no relationships were observed for either anti-MSP3 or anti-GLURP IgG levels and helminth status (Fig 3B and 3C).

### Helminth status and protection from malaria

**Fig 4:**
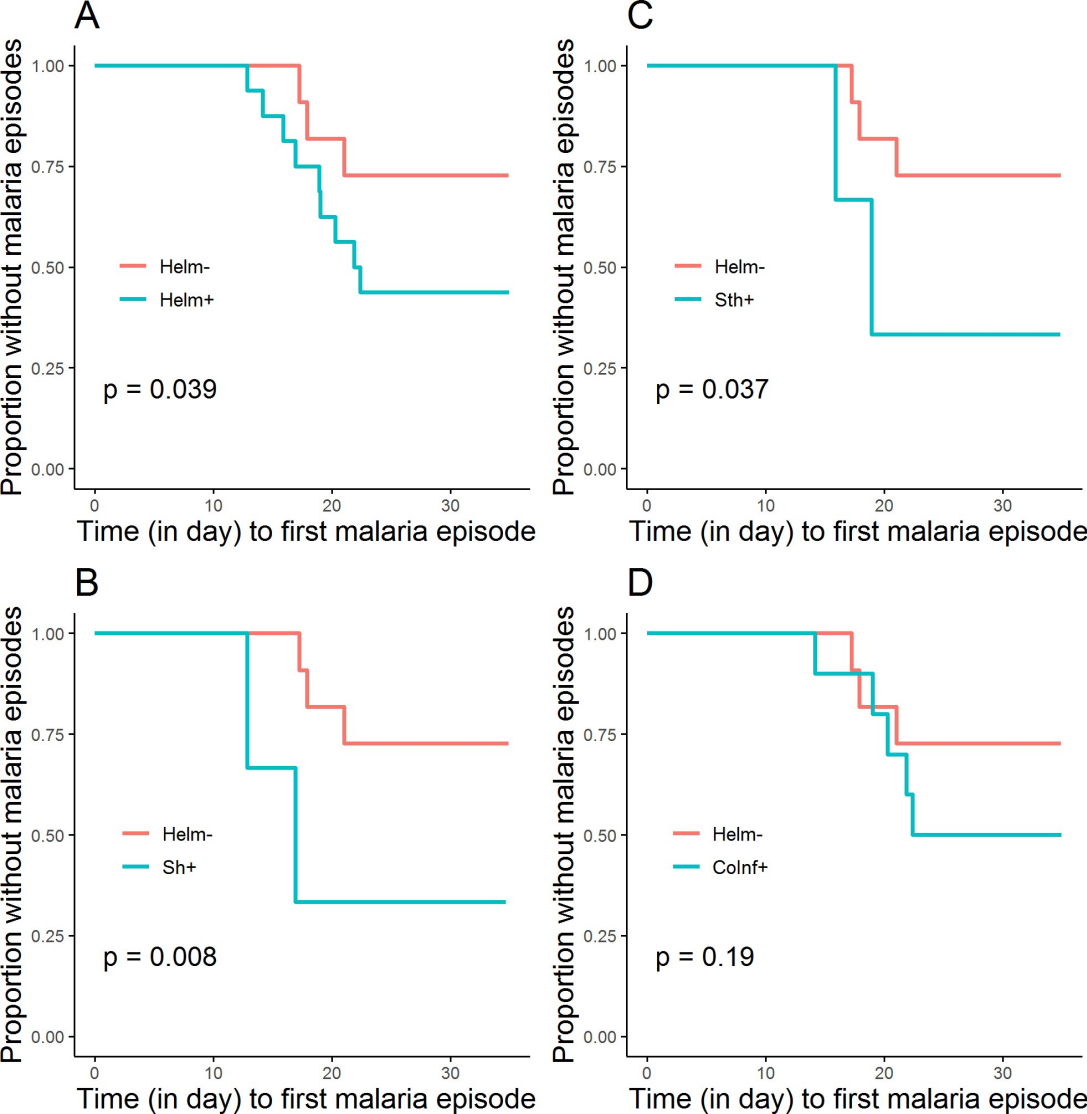
Time to occurrence of malaria episodes after CHMI.

Assessing the time to the occurrence of malaria episodes after CHMI between helminth groups, we found a relationship between any helminth infection and time to develop malaria (Log-Rank test: *p-value* = 0.039), as depicted in Fig 4A, such that those with helminth infection developed malaria significantly earlier than those without. When considering the infected subgroups, *S*. *haematobium* (Log-Rank test: *p-value* = 0.008) and STH (Log-Rank test: *p-value* = 0.037) infections were associated with faster times to malaria (Fig 4B and 4C). No statistically significant difference was observed between coinfection status and time to develop malaria (Log-Rank test: *p-value* = 0.19, Fig 4D).

The proportion of participants who did not develop malaria after CHMI is presented for Helm- (8/11, 72.7%) and Helm+ (7/16, 43.8%) groups (A), for Helm- and Sh+ (1/3, 33.3%) (B), for Helm- and Sth+ (1/3, 33.3%) (C), and in Helm- and CoInf+ (5/10, 50%) (D).

In additional analyses in the helminth coinfected group assessing the distribution of helminths species between malaria outcome groups following CHMI, the results showed that the distribution of helminth species was different between participants who did and those who did not develop malaria (S4 Table).

### Vaccine-specific IgG level and CHMI outcome according to helminth status

**Fig 5:**
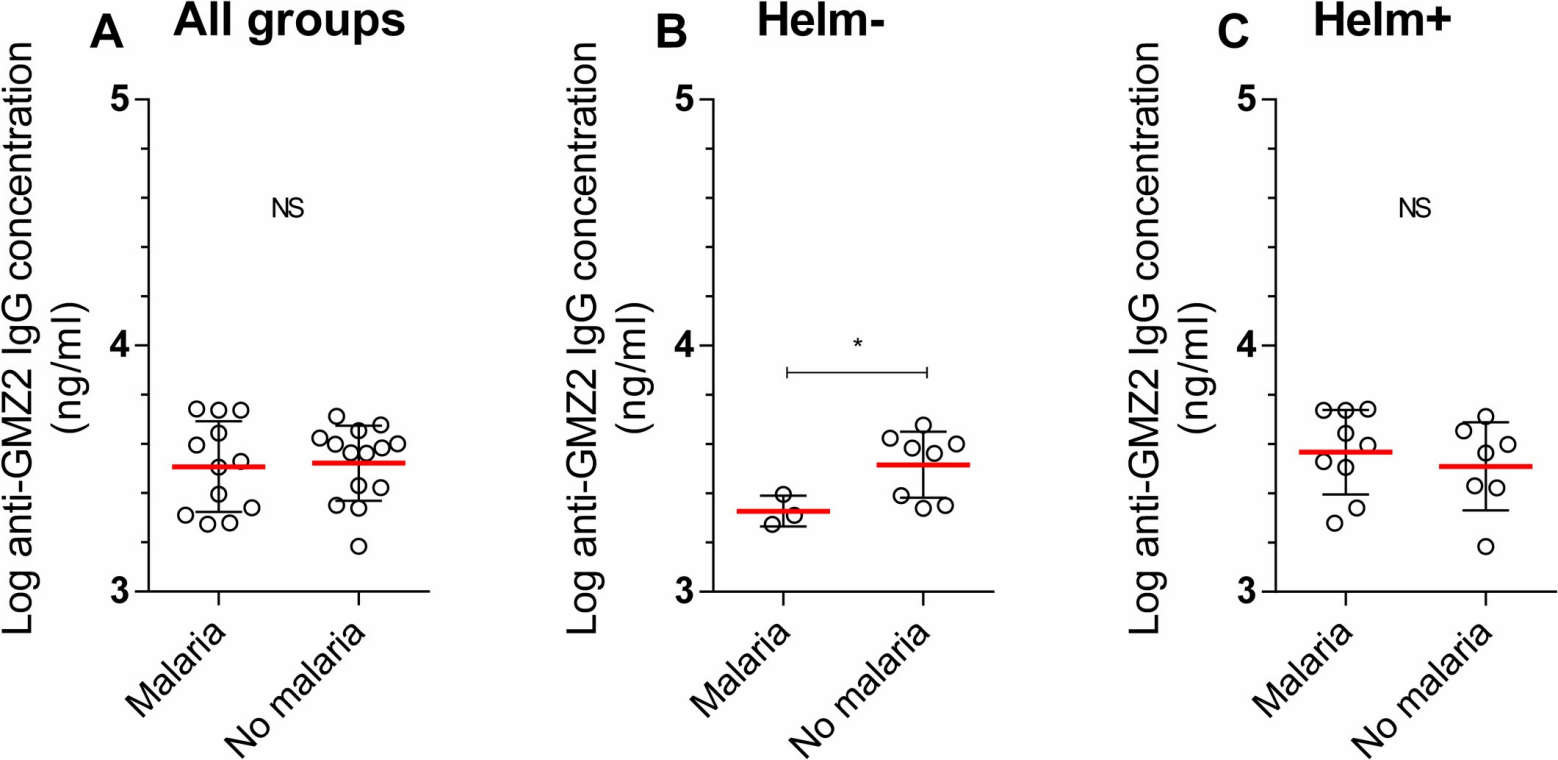
Anti-GMZ2 IgG concentration and CHMI outcome groups according to helminth status Comparison of vaccine-specific total IgG concentration at D84 between those having clinical malaria (monotone increase of parasitemia with symptoms) and those without clinical malaria (low oscillating parasitemia and no symptoms (control) and those with no parasitemia and no symptoms (protected)) after the CHMI was performed in all participants (n = 12 *vs* 14, 5A), in helminth uninfected (n = 3 *vs* 8, 5B), and infected (n = 9 *vs* 7, 5C) participants. The comparison was done by multivariate linear regression using baseline vaccine-specific total IgG concentration as variable of adjustment. Statistical significance was set for p value below 0.05. *p<0.05 NS = Non-significant.

As mentioned elsewhere [27], and confirmed here (Fig 5A), no difference was found between vaccine-specific IgG concentration on D84 and protection against clinical malaria following CHMI. We therefore assessed whether this observation differed according to helminth infection status. In a stratified analysis, a relationship was found between the occurrence of clinical malaria after CHMI and the anti-GMZ2 IgG concentration (*p-value* = 0.048) on D84 in the Helm- group. The anti-GMZ2 IgG concentration was higher among those who did not experience malaria (3.51 ±0.13) compared to those who experienced malaria (3.32 ±0.0.6, Fig 5B), although the increase was not statistically significant when adjusted with baseline anti-GMZ2 IgG concentration. In the Helm+ group, no such relationship was found (*p-value* = 0.51, Fig 5C).

## Discussion

The results of the study presented here show that helminth species can differentially affect the specific immune response following administration of a malaria vaccine candidate: participants infected with *S*. *haematobium* had higher GMZ2-specific IgG on D84 post-first immunization, while *S*. *stercoralis* infection was associated with a lower IgG response on D84 compared to that of helminth uninfected participants.

From our analysis, in comparison to helminth uninfected individuals, *S*. *haematobium* infection was associated with a higher post-immunization anti-GMZ2 IgG response. It has been shown that *S*. *haematobium* infection is associated with an increased systemic concentration of the C3d molecule of the complement system [41], which can enhance B cell signaling [42]. We speculate that this or a similar adjuvant-like phenomenon may explain our findings. Moreover, *S*. *haematobium* could augment antibody production by influencing the biological environment through enhanced IL-4 production [6], favoring antimalarial antibody production [43]. It should be noted that *S*. *haematobium* infection had no significant effect on the antibody responses directed to either MSP3 or GLURP, the two antigens combined in GMZ2, in contrast to the findings of a recent study [44], although the level of anti-GLURP IgG concentration was indeed higher in *S*. *haematobium* infected compared to uninfected individuals, but this difference was not statistically significant. It is well known that MSP3 constitutes a less immunogenic component of GMZ2 [24,25], possibly explaining this observation. The intensity of *S*. *haematobium* infection may have a determining role in its effect on other parasites. In our study cohort, the *S*. *haematobium* egg count in infected participants was low (S5 Table), possibly explaining the absence of any deleterious effect on the immunogenicity of GMZ2.

In contrast to our observations in *S*. *haematobium-*infected individuals, we observed low post-immunization anti-GMZ2 IgG levels in *S*. *stercoralis*-infected participants compared to post-immunization anti-GMZ2 IgG concentration in the helminth uninfected group. *S*. *stercoralis* has been shown to reduce B cell numbers, and to affect B cell responses during latent tuberculosis infection [45]. *S*. *stercoralis* can also reduce the induction of mycobacterial-specific IgM and IgG responses and the expression levels of the B-cell growth factors APRIL and BAFF [45]. There is virtually no data on the effect of this parasite on the immunogenicity of malaria vaccine antigens. In the present study, we show that this STH can negatively affect the vaccine IgG concentration as we have shown in a recent study conducted in children. We hypothesize that the presence of *S*. *stercoralis* in our study population could considerably inhibit B cell activity, via, for example, the induction of specific regulatory mechanisms including Treg [46].

In our study, co-infected participants had a similar specific IgG concentration compared to those uninfected, indicating potentially opposing effects of different species, possibly explaining the lower concentration of anti-GMZ2 IgG in the co-infected group compared to those harbouring *S*. *haematobium* alone; the question thus arises as to the importance of considering the effect of every single species of helminths separately on GMZ2 immunogenicity. Indeed, Esen and colleagues showed that infection with *T*. *trichiura* affects the concentration of anti-GMZ2 IgG in children, whilst infection with *A*. *lumbricoides* had no such effect [20], strongly suggesting a species-dependent effect.

Investigating the delay to development of malaria episodes with regard to helminth status, we observed that volunteers infected with helminths, irrespective of the species, develop malaria episodes earlier than those without these infections, possibly essentially due to *S*. *haematobium* and STH. Such an effect of *S*. *haematobium* and STH has been observed in the same area in young children [11], suggesting that helminths could affect malaria vaccine efficacy in adults through various mechanisms neither well known nor well described. We hypothesize that this effect is helminth species dependent. However, given the relatively small number of helminth mono-infected volunteers and the comparatively higher number of coinfected volunteers we had, it is difficult to elucidate any one infection’s role on susceptibility to malaria. Indeed, the absence of such an effect in co-infected volunteers supports the idea of opposing effects of these different helminth species, although the differences observed in the proportions of different helminths species between those who did and did not developed malaria after CHMI in the helminth coinfected could also explain the fact.

We found that those with no helminth infection who had a higher vaccine-specific IgG response on D84 were protected against clinical malaria. Antibodies are known to play an important role in acquired protective immunity against blood-stage malaria either through inhibition of merozoite invasion of red blood cells or antibody-mediated cell cytotoxicity against infected red blood cells [47,48]. From this standpoint, we hypothesize that the presence of helminths could affect the quality of the antibodies produced in response to the vaccine candidate, as described elsewhere [49,50], influencing their effect on *P*. *falciparum*. We speculate that the lack of GMZ2 efficacy shown in our study may therefore at least be partly due to the presence of helminths.

We recognize limitations to this study, notably the small number of infected individuals in the subgroups, precluding further in-depth statistical tests. In addition, the study was conducted exclusively on males, precluding the extrapolation of any conclusions to the population at large. The observational and exploratory nature of the study also means that causality cannot be easily addressed. Furthermore, the helminth groups are not randomized and likely differ systematically in other variables that may introduce bias. Another possible limitation is the CHMI, since it has never been used before in this context. Additionally, the fact that not all the available plasma at different time points were analyzed, and that the helminth diagnosis was not made between D0 and D84, weakens the results, because it would allow to check if these same observations are made at other time points. Despite these limitations, we did find some relevant and significant results and we believe that our findings provide important information on the negative effect of helminth infection on vaccine-induced protective immunity, and a probable double impact that helminths and *S*. *haematobium* in particular, could have on other parasites [11] or on the response to vaccine antigens. Other studies on the effect of helminths in malaria vaccine candidates on larger cohorts are needed to further corroborate these findings.

In summary, we report that the effect of helminth infection status on anti-GMZ2 immunogenicity could be parasite species-dependent, and that vaccine efficacy can be altered by helminth infections. If confirmed in other large cohort studies, it would clearly be therefore highly relevant to take into account volunteers’ helminth status when assessing the immunogenicity and efficacy of malaria vaccine candidates. A conclusion of this understanding may be to only include participants free of helminths, particularly in the early stages of clinical development of vaccines in which small sample sizes are a requisite.

## Supporting information

S1 Table Oligonucleotides List for the real time amplification. BHQ: Black hole quencher All the primers and probes were ordered by Eurofines. For the amplification we always did a triplex.
(DOCX)

S2 Table PCR mix optimized for our Study. Different primers and probes concentrations were tested to evaluate the best one for our assay. We used the following primers and probes concentration for each species: 0.1 μM, 0.2 μM, 0.3 μM and 0.4 μM. After checking and analyzing the PCR amplification curves, we decided to use the 0.2 μM concentration. Table 2 shows the chosen setting for our assay. # Volume depending of the reagent start concentration.
(DOCX)

S3 Table Proportion of helminth species in coinfected groups at D0 and/or at D84. Infection (+) either by PCR and/or microscopy at D0 and/or at D84 No infection (-) either by PCR and/or microscopy at D0 and/or at D84.
(DOCX)

S4 Table Distribution of helminth species in the helminth co-infected group in individuals who did or did not develop malaria following CHMI. The percentage of each helminth specie in the helminth coinfected group in those who did and did not develop malaria was calculated following the formula: (Number of volunteers infected at D0 and/or D84 x 100) / Total number of volunteers who did (Malaria) or did not develop malaria (No malaria) following CHMI.
(DOCX)

S5 Table *Schistosoma haematobium* egg counts per subject in infected volunteers. (DOCX)

S1 Fig Timeline of immunizations and blood sample collection I, II and III represent respectively the first, second and the third immunization. Blood collection was done before each immunization and at seven, fourteen days after the first and second immunization, and seven, fourteen and twenty-eight days after the third immunization, as well as one day before the CHMI and thirty-five day after the CHMI. The asterisk represents the time of urine and stool collection.
(TIF)

S2 Fig Anti-GMZ2, anti-MSP3 and anti-GLURP IgG concentrations between D0 and D84 in vaccination groups Figures show the log of GMZ2-specific (A), MSP3-specific (B), GLURP-specific (C) IgG concentrations at D0 (filled dot) and at D84 (unfilled dot) in participants vaccinated with Rabies vaccine, 100 μg GMZ2-Alhydrogel, 30 μg GMZ2-CAF01; and 100 μg GMZ2-CAF01. The comparison was performed using a paired t-test. The graphs show mean ± standard deviation. Statistical significance was set for p value below 0.05 *p<0.05, **p<0.01, ***p<0.001, ****p<0.0001. NS = Non-significant.
(TIF)

S1 Data Data underlying the findings. (XLSX)
