# Supplementary material for: Exploratory analysis of the effect of helminth infection on the immunogenicity and efficacy of the asexual blood-stage malaria vaccine candidate GMZ2
Source: PLoS Negl Trop Dis. 2021 Jun 1;15(6):e0009361. doi: 10.1371/journal.pntd.0009361 (PMC8195366; doi:10.1371/journal.pntd.0009361)
Supplement: S1 Table — BHQ: Black hole quencher All the primers and probes were ordered by Eurofines. For the amplification we always did a triplex. (DOCX) [file pntd.0009361.s001.docx]

**S1 Table**. Oligonucleotides List for the real time amplification

| **Target organism** | **GenBank accession No or References** | **Sequences** |
| --- | --- | --- |
| *Ascaris lumbricoides* | AJ000895 | Fwd Primer: GTAATAGCAGTCGGCGGTTTCTT  Rev Primer: GCCCAACATGCCACCTATTC  Probe: Texas Red-TTGGCGGACAATTGCATGCGAT-BHQ2 |
| *Strongyloides stercoralis* | AF279916 | Fwd Primer: GAATTCCAAGTAAACGTAAGTCATTAGC  Rev Primer: TGCCTCTGGATATTGCTCAGTTC  Probe: Texas Red-ACACACCGGCCGTCGCTGC-BHQ2 |
| *Necator americanus* | AJ001599 | Fwd Primer: CTGTTTGTCGAACGGTACTTGC  Rev Primer: ATAACAGCGTGCACATGTTGC  Probe: FAM-CTGTACTACGCATTGTATAC-BHQ1 |
| *Trichuris trichiura* | AB699091 | Fwd Primer: TTGAAACGACTTGCTCATCAACTT  Rev Primer: CTGATTCTCCGTTAACCGTTGTC  Probe:FAM-CGATGGTACGCTACGTGCTTACCATGG-BHQ1   \|  \|  \| \| --- \| --- \| |
| *Phocin herpes virus(PhHV)* | [34] | Fwd Primer: GGGCGAATCACAGATTGAATC  Rev Primer: GCGGTTCCAAACGTACCAA  Probe: CY5 -TTTTTATGTGTCCGCCACCATCTGGATC-BHQ2 |

BHQ: Black hole quencher

All the primers and probes were ordered by Eurofines. For the amplification we always did a triplex.
