# Supplementary material for: Exploratory analysis of the effect of helminth infection on the immunogenicity and efficacy of the asexual blood-stage malaria vaccine candidate GMZ2
Source: PLoS Negl Trop Dis. 2021 Jun 1;15(6):e0009361. doi: 10.1371/journal.pntd.0009361 (PMC8195366; doi:10.1371/journal.pntd.0009361)
Supplement: S2 Table — Different primers and probes concentrations were tested to evaluate the best one for our assay. We used the following primers and probes concentration for each species: 0.1 μM, 0.2 μM, 0.3 μM and 0.4 μM. After checking and analyzing the PCR amplification curves, we decided to use the 0.2 μM concentration. Table 2 shows the chosen setting for our assay. # Volume depending of the reagent start concentration. (DOCX) [file pntd.0009361.s002.docx]

**S2 Table.** PCR mix optimized for our Study

| Reagents | Concentration | Volume |
| --- | --- | --- |
|  |  |  |
| 2x Qiagen QuantiTect Multiplex PCR NoROX | (1x) | 10 µL |
|  |  |  |
| 10mg/ml BSA (New England Biolabs) | (0,1mg/ml) | 0,2 µL |
|  |  |  |
| Forward primer 1 | (0,2 µM) | # |
| Reverse primer 1 | (0,2 µM) | # |
| Probe 1(FAM) | (0,2 µM) | # |
|  |  |  |
| Forward primer 2 | (0,2 µM) | # |
| Reverse primer 2 | (0,2 µM) | # |
| Probe 2(Texas Red) | (0,2 µM) | # |
|  |  |  |
| Forward primer 3 | (0,2 µM) | # |
| Reverse primer 3 | (0,2 µM) | # |
| Probe 3(Cy5) | (0,2 µM) | # |
|  |  |  |
| Template DNA | x | 5 µL |
| RNase free water | x | up to 20 µL |
|  |  |  |
| Total reaction volume |  | 20 µL |

Different primers and probes concentrations were tested to evaluate the best one for our assay. We used the following primers and probes concentration for each species: 0.1 µM, 0.2 µM, 0.3 µM and 0.4 µM. After checking and analyzing the PCR amplification curves, we decided to use the 0.2 µM concentration. The table 2 shows the chosen setting for our assay.

# Volume depending of the reagent start concentration
