# Supplementary material for: Exploratory analysis of the effect of helminth infection on the immunogenicity and efficacy of the asexual blood-stage malaria vaccine candidate GMZ2
Source: PLoS Negl Trop Dis. 2021 Jun 1;15(6):e0009361. doi: 10.1371/journal.pntd.0009361 (PMC8195366; doi:10.1371/journal.pntd.0009361)
Supplement: S3 Table — Infection (+) either by PCR and/or microscopy at D0 and/or at D84 No infection (-) either by PCR and/or microscopy at D0 and/or at D84. (DOCX) [file pntd.0009361.s003.docx]

**S3 Table.** Proportion of helminth species in coinfected groups at D0 and/or at D84

| Participant identification | Helminth species | | | | |
| --- | --- | --- | --- | --- | --- |
|  | *S.* *stercoralis* | *A.* *lumbricoides* | Hookworm | *S.* *haematobium* | *T. trichiura* |
| 1 | + | + | - | - | - |
| 2 | - | - | + | + | - |
| 3 | - | - | + | + | + |
| 4 | + | - | - | - | + |
| 5 | - | - | - | + | + |
| 6 | - | - | + | + | + |
| 7 | - | - | - | + | + |
| 8 | + | - | + | + | - |
| 9 | - | - | + | + | + |
| 10 | - | - | - | + | + |
| 11 | - | - | + | + | - |
| 12 | - | + | - | + | + |
| Total (%) | 3 (10.3) | 2 (6.9) | 6 (20.7) | 10 (34.5) | 8 (27.6) |

Infection (+) either by PCR and/or microscopy at D0 and/or at D84

No infection (-) either by PCR and/or microscopy at D0 and/or at D84.
