# Supplementary material for: Exploratory analysis of the effect of helminth infection on the immunogenicity and efficacy of the asexual blood-stage malaria vaccine candidate GMZ2
Source: PLoS Negl Trop Dis. 2021 Jun 1;15(6):e0009361. doi: 10.1371/journal.pntd.0009361 (PMC8195366; doi:10.1371/journal.pntd.0009361)
Supplement: S4 Table — The percentage of each helminth specie in the helminth coinfected group in those who did and did not develop malaria was calculated following the formula: (Number of volunteers infected at D0 and/or D84 x 100) / Total number of volunteers who did (Malaria) or did not develop malaria (No malaria) following CHMI. (DOCX) [file pntd.0009361.s004.docx]

**S4 Table**: Distribution of helminths species in helminth co-infected group in individuals who did or did not develop malaria after CHMI

| **Helminths species** | **Number of helminth infected at D0 and/or at D84 (%)** | |
| --- | --- | --- |
|  | **Malaria (n=5)** | **No malaria (n=5)** |
| **Hookworm** | 3 (60) | 2 (40) |
| ***A. lumbricoides*** | 3 (60) | 0 (0) |
| ***S. haematobium*** | 3 (60) | 5 (100) |
| ***S. stercoralis*** | 2 (40) | 2 (40) |
| ***T. trichiura*** | 3 (60) | 5 (100) |

The percentage of each helminth specie in the helminth coinfected group in those who did and did not develop malaria was calculated following the formula: (Number of volunteers infected at D0 and/or D84 x 100) / Total number of volunteers who did (Malaria) or did not develop malaria (No malaria) following CHMI.
