# Supplementary material for: Exploratory analysis of the effect of helminth infection on the immunogenicity and efficacy of the asexual blood-stage malaria vaccine candidate GMZ2
Source: PLoS Negl Trop Dis. 2021 Jun 1;15(6):e0009361. doi: 10.1371/journal.pntd.0009361 (PMC8195366; doi:10.1371/journal.pntd.0009361)
Supplement: S5 Table — (DOCX) [file pntd.0009361.s005.docx]

**S5 Table**: *Schistosoma haematobium* eggs counts per subject in infected volunteers

| Patient identification | *S. haematobium* eggs Counts (per 10ml of urine) at D0 | *S. haematobium* eggs Counts (per 10ml of urine) at D84 |
| --- | --- | --- |
| 1 | 1 | 0 |
| 2 | 0 | 6 |
| 3 | 7 | 0 |
| 4 | 7 | 0 |
| 5 | 9 | 2 |
| 6 | 1 | 12 |
| 7 | 0 | 17 |
| 8 | 11 | 32 |
| 9 | 0 | 49 |
| 10 | 1 | 50 |
| 11 | 53 | 0 |
| 12 | 35 | 50 |
| 13 | 179 | 51 |
| 14 | 240 | 0 |
