# Supplementary figures and images for: Exploratory analysis of the effect of helminth infection on the immunogenicity and efficacy of the asexual blood-stage malaria vaccine candidate GMZ2
Source: PLoS Negl Trop Dis. 2021 Jun 1;15(6):e0009361. doi: 10.1371/journal.pntd.0009361 (PMC8195366; doi:10.1371/journal.pntd.0009361)

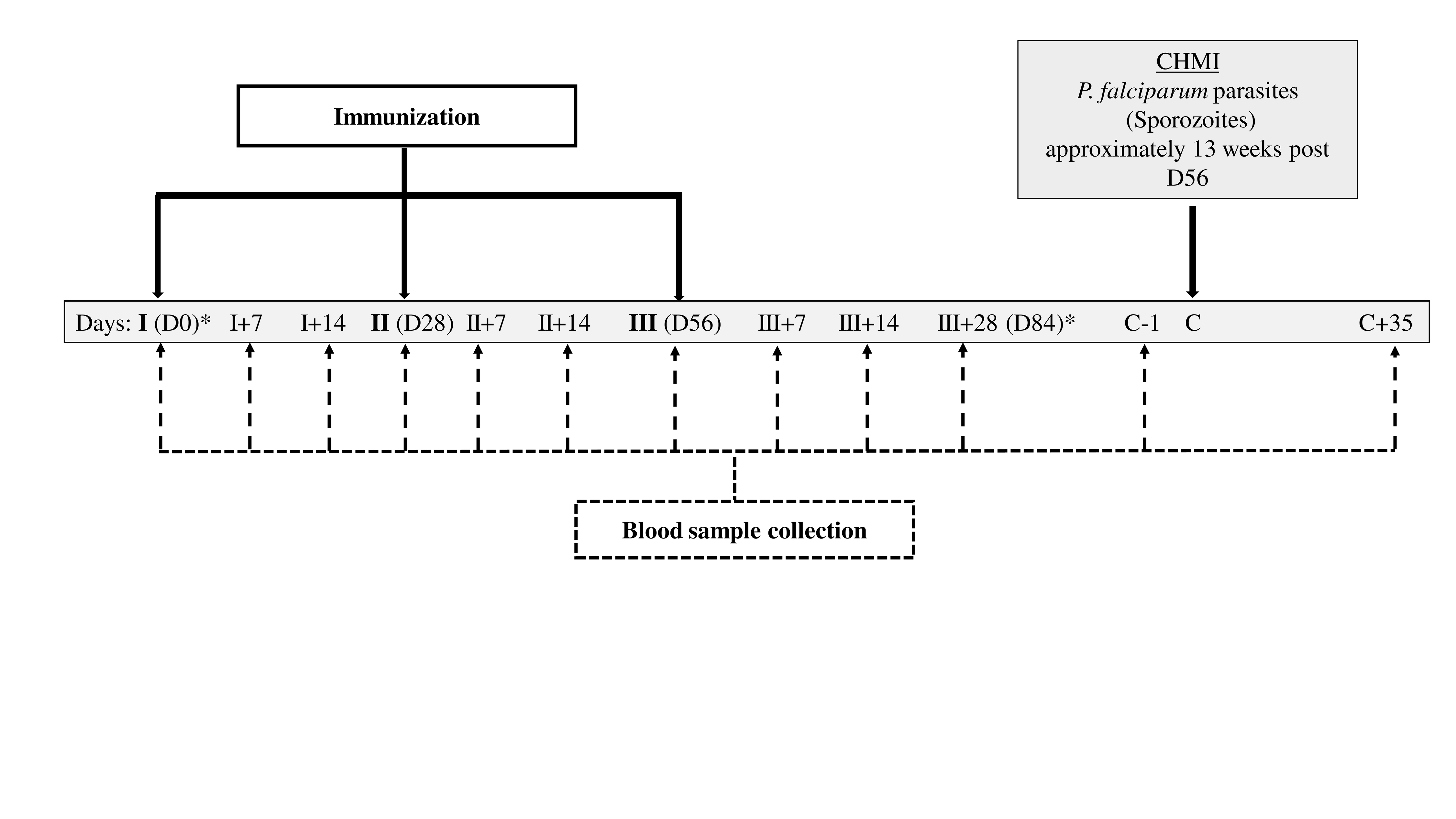

Supplement: S1 Fig — Blood collection was done before each immunization and at seven, fourteen days after the first and second immunization, and seven, fourteen and twenty-eight days after the third immunization, as well as one day before the CHMI and thirty-five day after the CHMI. The asterisk represents the time of urine and stool collection. (TIF) [file pntd.0009361.s006.tif]

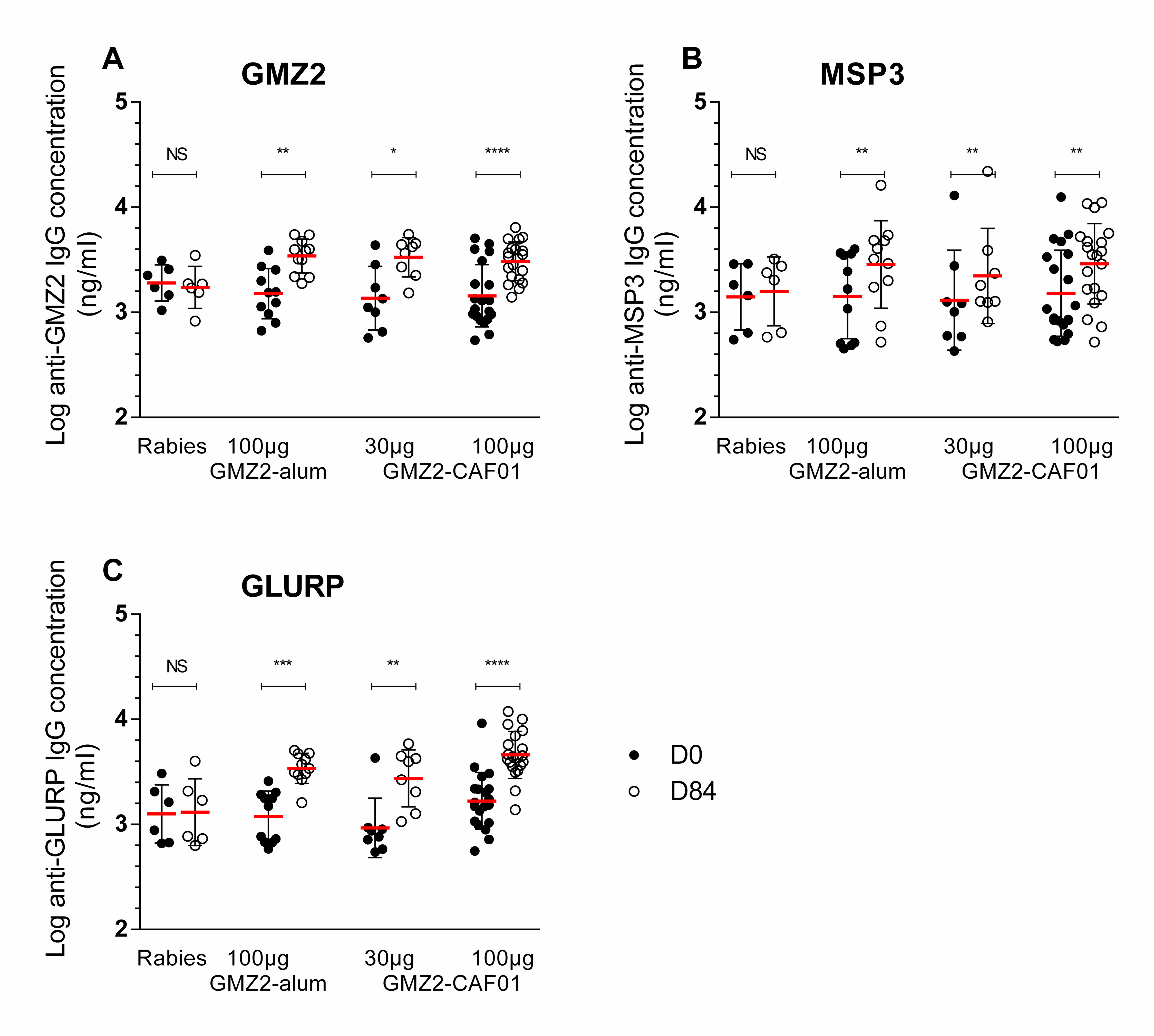

Supplement: S2 Fig — Anti-GMZ2, anti-MSP3 and anti-GLURP IgG concentrations between D0 and D84 in vaccination groups Figures show the log of GMZ2-specific (A), MSP3-specific (B), GLURP-specific (C) IgG concentrations at D0 (filled dot) and at D84 (unfilled dot) in participants vaccinated with Rabies vaccine, 100 μg GMZ2-Alhydrogel, 30 μg GMZ2-CAF01; and 100 μg GMZ2-CAF01. The comparison was performed using a paired t-test. The graphs show mean ± standard deviation. Statistical significance was set for p value below 0.05 *p<0.05, **p<0.01, ***p<0.001, ****p<0.0001. NS = Non-significant. (TIF) [file pntd.0009361.s007.tif]
